# Use of Palliative Chemotherapy and ICU Admissions in Gastric and Esophageal Cancer Patients in the Last Phase of Life: A Nationwide Observational Study

**DOI:** 10.3390/cancers13010145

**Published:** 2021-01-05

**Authors:** Joost Besseling, Jan Reitsma, Judith A. Van Erkelens, Maike H. J. Schepens, Michiel P. C. Siroen, Cathelijne M. P. Ziedses des Plantes, Mark I. van Berge Henegouwen, Laurens V. Beerepoot, Theo Van Voorthuizen, Lia Van Zuylen, Rob H. A. Verhoeven, Hanneke van Laarhoven

**Affiliations:** 1 Department of Medical Oncology, Amsterdam UMC, Cancer Center Amsterdam, University of Amsterdam, 1081 HV Amsterdam, The Netherlands; c.vanzuylen@amsterdamumc.nl (L.V.Z.); r.verhoeven@iknl.nl (R.H.A.V.); 2 Zorgverzekeraars Nederland, 3708 JE Zeist, The Netherlands; j.reitsma@zn.nl (J.R.); m.schepens@zn.nl (M.H.J.S.); 3 Vektis, 3708 JE Zeist, The Netherlands; j.van.erkelens@vektis.nl; 4 CZ Zorgverzekeringen, 5038 KE Tilburg, The Netherlands; michiel.siroen@cz.nl; 5 Zilveren Kruis, 3833 LB Leusden, The Netherlands; cathelijne.ziedses.des.plantes@zilverenkruis.nl; 6 Department of Surgery, Amsterdam UMC, Cancer Center Amsterdam, University of Amsterdam, 1081 HV Amsterdam, The Netherlands; m.i.vanbergehenegouwen@amsterdamumc.nl; 7 Elisabeth-TweeSteden Hospital, 5042 AD Tilburg, The Netherlands; l.beerepoot@etz.nl; 8 Rijnstate Hospital, 6815 AD Arnhem, The Netherlands; tvanvoorthuizen@rijnstate.nl; 9 Department of Research & Development, Netherlands Comprehensive Cancer Organisation, 3511 DT Utrecht, The Netherlands

**Keywords:** gastroesophageal cancer, palliative care, nationwide, end of life

## Abstract

**Simple Summary:**

This is the first nationwide study on chemotherapy use and intensive care unit (ICU) admission in the last three months before death in patients with cancer of the stomach or esophagus. Chemotherapy use and ICU admission shortly before death were relatively infrequent in the Netherlands. Chemotherapy was used less often in hospitals that treat many patients compared to hospitals that treat fewer patients. In patients that received chemotherapy before their final three months before death, chemotherapy was prescribed four times more often in the last three months before death compared to patients without previous chemotherapy use.

**Abstract:**

Since intensive care unit (ICU) admission and chemotherapy use near death impair the quality of life, we studied the prevalence of both and their correlation with hospital volume in incurable gastroesophageal cancer patients as both impair the quality of life. We analyzed all Dutch patients with incurable gastroesophageal cancer who died in 2017–2018. National insurance claims data were used to determine the prevalence of ICU admission and chemotherapy use (stratified on previous chemotherapy treatment) at three and one month(s) before death. We calculated correlations between hospital volume (i.e., the number of included patients per hospital) and both outcomes. We included 3748 patients (mean age: 71.4 years; 71.4% male). The prevalence of ICU admission and chemotherapy use were, respectively, 5.6% and 21.2% at three months and 4.2% and 8.0% at one month before death. Chemotherapy use at three and one months before death was, respectively, 4.3 times (48.0% vs. 11.2%) and 3.7 times higher (15.7% vs. 4.3%), comparing patients with previous chemotherapy treatment to those without. Hospital volume was negatively correlated with chemotherapy use in the final month (r_weighted_ = −0.23, *p* = 0.04). ICU admission and chemotherapy use were relatively infrequent. Oncologists in high-volume hospitals may be better equipped in selecting patients most likely to benefit from chemotherapy.

## 1. Introduction

Gastric and esophageal cancer are the sixth and ninth most prevalent cancers worldwide [1]. They account for 8.2% and 5.3% of cancer-related mortality, respectively [2,3]. This high mortality rate is partly explained by the relatively large proportion of patients (30% to 40%) with stage IV cancer at the time of diagnosis [4,5]. Curative treatment options in these patients are unavailable, and consequently, care focuses on symptom relief. Palliative chemotherapy can play a role in the treatment of these patients by extending lifetime while retaining the quality of life [6]. However, in a palliative setting near the end of life, aggressive treatment, such as intensive chemotherapy or admission to an intensive care unit (ICU), is often not appropriate, as these treatments are associated with depression and worse quality of life for patients and family caregivers [7]. Consequently, the costs of medical care will increase, without apparent benefit. On the other hand, interventions aimed to reduce aggressive treatment near the end of life are associated with an improved quality of life [8,9,10]. In addition, considering the strong rising health care expenditures, expensive treatments without apparent benefit should be critically reviewed.

The use of chemotherapy and ICU admission in the last phases of life are associated with younger age, male gender, non-white ethnicity, lower cancer stage, and the presence of comorbidities [11,12]. It has also been suggested that there might be a correlation between ICU admission and type of hospital (small, community, or teaching) [11]. For high-volume hospitals, longer survival times have been reported for patients with gastric and esophageal cancer compared to lower-volume hospitals [13]. Although case-mix factors cannot be excluded, a higher level of experience in health care workers, which comes with increased exposure to this patient population, may also contribute to these findings. Therefore, it may be hypothesized that this same level of experience could result in a more balanced use of aggressive treatment near the end of life.

Only a few publications on the use of chemotherapy or ICU admissions near the end of life in patients with gastric or esophageal cancer are available. In these studies, chemotherapy use varied from 8% in the last month before death [11] to 36% in the final three months [14]. Admission to an ICU in the last month occurred in 6% to 19% of patients [11,12], and these proportions appeared to be higher in patients that previously received palliative chemotherapy [15]. However, these studies are subject to varying limitations: e.g., they were conducted in only one region [11] or a single hospital [15], patients were selected for having one specific health care insurance plan [14], or the study focused only on in-hospital deaths among metastasized patients [12] or grouped all cancers of the digestive tract together [11].

To address these issues, we conducted an observational, nationwide study in the Netherlands in all deceased patients with gastric and esophageal cancer to answer the following questions: (1) to what extent are patients treated with chemotherapy and/or admitted to the ICU in their final months; (2) to what extent is chemotherapy continued near the end of life; (3) to what extent are these treatment decisions related to the number of experience of the healthcare institutions?

## 2. Methods

### 2.1. Study Design and Population Selection

The research questions are answered using Dutch medical claims data. These data cover the whole Dutch population, containing claims of all patients, healthcare providers, and claims of delivered care from health care institutions to health care insurance companies. Demographic characteristics such as age and gender are also available. Claims include both prescribed medications as well as interventional procedures, including radiotherapy. The supplementary material (Tables S1–S6) describes the definitions that were used.

The research population for this study is defined as deceased patients diagnosed with gastric or esophageal cancer without curative treatment. We selected all deceased patients in 2017 and 2018 who had a diagnosis of gastric or esophageal cancer. Patients that received one or more potentially curative treatments were excluded from the population. These included: surgical resections, radiation in combination with chemotherapy, and endoscopic mucosal resection or endoscopic submucosal dissection. Chemoradiation was defined as at least fourteen radiation fractions with at least one chemotherapy treatment session within four days after the start of radiation. Patients that first received curative treatment and in a later stadium underwent palliative treatment were excluded as well since the structure of the data did not allow distinction between these patients and patients receiving curative treatment only. Treatment schemes with chemotherapy in the month before the start of radiation were regarded as palliative treatment.

### 2.2. Outcomes and Characteristics

Treatment of the palliative population in one (30 days) and three months (90 days) before death was studied with regard to ICU admission for at least one day and chemotherapy administration (at least one treatment session). For chemotherapy, analyses were stratified on chemotherapy treatment prior to the final month or final three months. This was done to detect a potential influence of previously started chemotherapy on the administration of chemotherapy near the end of life. Previous chemotherapy use was defined as chemotherapy initiated prior to the final month or final three months. We used hospital volume, defined as the number of patients that were included in the research population per institute, as a proxy for hospital experience. We hypothesize that increased hospital experience (i.e., the experience of hospital personnel with the studied patient population) is related to the improved distinction between patients that benefit from aggressive treatment and those who do not. Hospital volume was operationalized in two ways. First, the specific measure of hospital volume was defined as the number of patients per institution who met the inclusion criteria for this study and the specific indicators. This measure reflects hospital experience with patients in a palliative setting in the final months of life. Patients who were treated in more than one hospital were assigned to the hospital with the most declarations for treatment of gastroesophageal cancer in the two years before death (if there was an equal number of declarations, the last declaration was decisive). Second, to explore whether hospital experience with chemotherapy prescription per se is associated with the outcome indicators, we used a broader treatment experience measure not limited to deceased or palliative patients. For this broader experience measure, the volume of all gastroesophageal cancer patients that received systemic treatment in a curative setting or palliative systemic therapy was calculated, as described earlier [16]. With the aim to reflect current practice, the volume of recent years (2017 and 2018) was used. Age and gender were analyzed as background characteristics.

### 2.3. Statistical Analyses

Comparison of means was performed with independent sample *t*-tests, equal variances assumed. Comparison of percentages was performed with contingency tables and chi-square tests. The relation between the treatment and hospital experience was studied with correlation analyses at the hospital level.

The correlations were weighted, where the weight was the number of observations (patients) in order to avoid results that were highly influenced by institutional-level statistics by small patient number institutes. All reported statistical tests are two-tailed where *p* < 0.05 was considered to be statistically significant. All analyses were performed with SAS software (version 9.4, SAS Institue Lnc., Cary, NC, USA).

## 3. Results

### 3.1. Population

**Figure 1:**
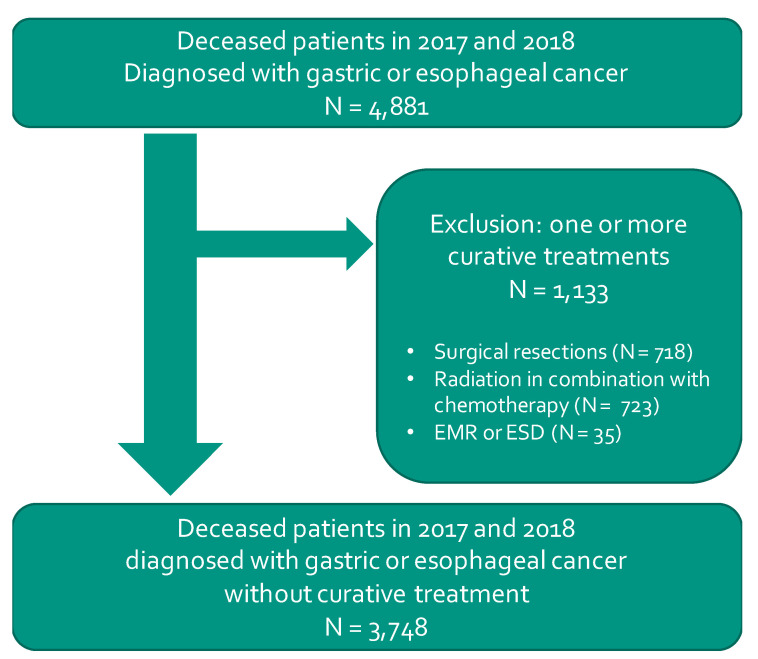
Flowchart of population definition.

Figure 1 shows a flowchart of the population definition. In total, 4881 patients diagnosed with gastric or esophageal cancer died in 2017 and 2018. Patients who received one or more curative treatments (in total *N* = 1133) were excluded from the population. The remaining population consisted of 3748 patients.

The average age at the time of death was 71.4 years (standard deviation (SD) = 11.1). Most patients were male (71.4%). Table 1 summarizes other descriptive statistics of the population of patients.

### 3.2. ICU Admission and Chemotherapy

Of all included patients, 5.6% were admitted to the ICU three months before death and 4.2% in the last month before death (Table 2). Chemotherapy was administered in 21.2% of patients in the last three months before death and 8% one month before death. Chemotherapy use at three months before death was more prevalent among patients that already received chemotherapy prior to the three months before their death (48.0%) compared to those that were treatment-naïve (11.2%; relative risk (RR) = 4.3; 95% confidence interval (CI) = 3.8–4.8). Similar results were found if chemotherapy use in the final month before death was the outcome of interest; this was more prevalent among patients that used chemotherapy prior to their last month (15.7%) compared to those that did not (4.3%; RR: 3.7; 95%CI = 2.9–4.6).

### 3.3. Outcomes in Relation to Age and Gender

Admission to the ICU was not significantly related to either age or gender. The average age in patients with and without ICU admission in their final three months was 70.2 years (standard deviation (SD) = 10.4) and 71.4 years (SD = 11.2; *p* = 0.13), respectively. For ICU admission in the final month, we found a similar pattern: patients with and without ICU admission were, on average, 70.4 (SD = 9.9) and 71.4 (SD = 11.2) years old (*p* = 0.29)).

The percentage of males among patients with ICU admission in their final three months (69.9%) did not differ significantly from those without ICU admission in this period (71.5%; *p* = 0.61). The same holds for the final month (73.7% versus 71.3%; *p* = 0.51).

Patients receiving chemotherapy in their final three months were younger (mean age: 64.3 years, SD = 9.9) compared to patients who did not receive chemotherapy (mean age: 73.3 years, SD = 10.7; *p* < 0.001). For the final month, we found a similar pattern: mean age was 65.4 years (SD = 9.7) and 71.9 years (SD = 11.2; *p* < 0.001) in patients that, respectively, did and did not receive chemotherapy. Patients who received chemotherapy before their final three months were relatively young (63.5 years, SD = 10.0) compared to those who did not receive chemotherapy before (65.6 years, SD = 9.7; *p* = 0.004). This difference was not significant in the final month; patients who received chemotherapy previously (64.6 years, SD = 9.4) have a comparable mean age with the group without previous chemotherapy (66.6 years, SD = 10.0; *p* = 0.09).

Chemotherapy use in the final three months was more prevalent among males compared to females (22.8% versus 17.3%; *p* < 0.001). In the final month, the difference in chemotherapy use is borderline significant (8.5% in males and 6.6% in females; *p* = 0.05).

### 3.4. Outcomes in Relation to Hospital Experience

First, we studied the relation of ICU admission and chemotherapy treatment with the specific hospital experience measure for these indicators (Table 3). We did not find a significant correlation between the specific hospital experience and ICU admission in the last three months (r_weighted_ = 0.18, *p* = 0.11), but the correlation was borderline significant for chemotherapy use in the final month before death (r_weighted_ = 0.20, *p* = 0.07). We found no correlation between specific hospital experience and chemotherapy use in the final three months before death (r_weighted_ = −0.12, *p* = 0.29). However, there was a significant negative correlation between hospital experience and chemotherapy use in the final month (r_weighted_ = −0.23; *p* = 0.04). This particularly holds for patients without previous chemotherapy in their final month (r_weighted_ = −0.23; *p* = 0.03) but not for patients with previous chemotherapy (r_weighted_ = −0.18; *p* = 0.12).

Second, we studied the relation of ICU admission and chemotherapy treatment with the broader hospital experience measure (Table 4). This broader definition of experience was not significantly correlated with ICU admission or chemotherapy use in patients’ final months. However, within the subgroup of patients who were treated previously to their final three months of life, we found a significant negative correlation with chemotherapy in the final three months before death (r = −0.23; *p* = 0.04).

## 4. Discussion

### 4.1. Main Findings

In this nationwide, observational study in 3748 deceased patients with gastric and esophageal cancer, we found that chemotherapy was used by, respectively, 21.2% and 8.0% of patients at three months and one month before death. Chemotherapy use in the final three months was about four times higher among patients with prior use of chemotherapy compared to those without. Admission to the ICU in the three months or last month before death occurred in 5.6% and 4.2% of patients, respectively. Furthermore, we found a significant negative correlation between the number of palliative patients in the hospital during the last phase of their life and the use of chemotherapy in a patient’s last month. This particularly holds for patients not previously treated with chemotherapy. Using the prescription of chemotherapy in both the curative and palliative patients as an experience indicator, a significant negative correlation with chemotherapy use was found for patients in the final three months of life who were treated with chemotherapy before. There was no correlation between this hospital experience indicator and ICU admissions.

### 4.2. Clinical Relevance

Compared to previously published data, the prevalence of chemotherapy use near the end of life is lower in the Netherlands than in other western countries such as Canada [11] and France [14]. Patient selection might explain this difference. The Canadian study included a broader group of patients (i.e., all patients with cancer of the digestive tract), in which indications for chemotherapy differ from patients with gastric or esophageal cancer only. These indications might result in prolonged use of chemotherapy near the end of life. The French study included in-hospital deaths only; it can be suspected that a substantial part of these patients was admitted due to an unforeseen event, and thus the cancellation of chemotherapy in anticipation of the end of life could not be realized.

In addition, in the Netherlands, doctors and patients presumably discuss the benefits and disadvantages of aggressive treatment near the end of life more frequently. This is exemplified by an international survey among general practitioners showing that treatment preferences near the end of life were discussed six times more often in the Netherlands compared to Italy and Spain and two times more often compared to Belgium [17]. The probability of in-hospital death was the lowest in the Netherlands in this study, which suggests patients pass away more often at home, according to their wish [18]. A US study showed that end-of-life discussions are associated with less aggressive treatment shortly before death [7]. Together, these findings support the interpretation that early discussion of end-of-life treatments in the Netherlands can contribute to the lower prevalence of chemotherapy use and ICU admission in our study.

Nevertheless, from our data follows that a substantial proportion of patients undergo treatment that is likely to lower their quality of life and does not contribute to their survival. However, to identify those patients in whom the discontinuation of chemotherapy might be beneficial can be challenging. In this conundrum, unplanned hospitalization of a patient can provide a helping hand; in patients with advanced cancer who receive chemotherapy, unplanned hospitalization is associated with a two to three times higher mortality [19]. Hospitalization therefore may flip the balance between advantages and disadvantages of chemotherapy to the latter. Unplanned hospitalizations or a following outpatient clinical appointment also seem to be natural moments to discuss the continuation of chemotherapy. Shared decision making in health care delivery is an important topic of discussion nowadays and may assist in making the right choice that is tailored to the expectations and wishes of each individual patient.

Based on our results, it may be hypothesized that oncologists may be subject to a phenomenon known as “sunk cost bias”; if a substantial amount of time, money, or effort is invested in a certain trajectory, physicians find it more difficult to suspend this trajectory, although objective arguments favor cancellation [20]. This can explain the finding that chemotherapy use in the final three months is four times higher among patients who previously received chemotherapy versus those who did not. Interestingly, in hospitals with a large number of both curative and palliative gastroesophageal cancer patients treated with systemic therapy, a lower number of patients that were previously treated with chemotherapy continued to receive chemotherapy in the last months of their lives. We found a negative correlation between the hospital volume of palliative patients and the prescription of chemotherapy in their final month. This could implicate that oncologists working in hospitals in which a high number of patients are treated (possibly resulting in more experience) are better able to define the patients that will not benefit from continuation chemotherapy in the last months of life. Two factors can possibly explain this phenomenon. Firstly, physicians in low-volume hospitals are exposed to patients for whom a treatment decision has to be made less often, resulting in a lower level of experience. It has been shown that more experienced doctors exhibit lower resource utilization, reflected by the length of hospital stay and health care cost per patient [21]. Secondly, previous research indicates that a multidisciplinary approach and sufficient choice in treatment options contribute to improved outcomes in surgical procedures [22]. These are more often present in high-volume centers. One might hypothesize that these attributes also improve the selection of patients eligible for palliative chemotherapy.

A method by which the experience and multidisciplinary approach of high-volume hospitals can be shared with low-volume hospitals is teleconsultation. In cancer patients, this already has been shown to reduce the rate of chemotherapy-related adverse events [23] and provide more comprehensive care [24]. During the recent COVID-19 outbreak, the implementation of interhospital teleconsultation between oncology departments in Italy was accelerated [25], emphasizing its value and feasibility. Another possibility in sharing knowledge is a regional cancer network in which multiple hospitals collaborate, whereby one high-volume hospital acts as an expert center where cases can be presented in a regional multidisciplinary team meeting, either live or by teleconsultation. This may facilitate high-quality health care delivery as close to the patient as possible.

### 4.3. Methodological Considerations

Some methodological aspects of our study merit discussion. First of all, due to its retrospective nature, it is subject to unmeasured confounders, particularly regarding the correlations we investigated. Comorbidities such as hypertension, diabetes, and COPD are associated with less use of chemotherapy in the last phase of life [11] and might have influenced our results. In particular, the correlation between hospital size and chemotherapy use is of interest, since high-volume hospitals can theoretically differ from low-volume hospitals regarding comorbidities of patients [26]. However, the association between comorbidities and chemotherapy use is weak (odds ratio = 0.94) [11], and in other studies from the Netherlands, the prevalence of comorbidities was equally distributed among high- and low-volume hospitals [27,28]. Therefore, we deem the potential influence of comorbidities on the reported correlations to be very low.

Secondly, patients were retrospectively selected for being deceased. Consequently, prospective indicators of aggressive treatment near the end of life could not be investigated. Caution is needed in drawing conclusions on causality with respect to the correlations we found. A prospective study would be helpful in answering these issues.

Thirdly, the selection procedure we applied possibly resulted in an underestimation of chemotherapy use by the incorrect exclusion of patients in a palliative setting. For example, patients that started a chemoradiotherapy plan that was deemed to be curative were excluded, even if this plan was terminated prematurely. The termination could have been motivated by the progression of the disease to stage IV cancer, whether by the natural course of the disease or new findings on imaging studies. If these patients were included in our study, the prevalence of chemotherapy use is likely to be higher. However, we feel the exclusion of these patients is justified in our study. Including patients that started a chemoradiotherapy plan that was deemed to be curative but terminated prematurely would probably result in the additional inclusion of patients in a curative setting. For these patients, no distinction can be made between those patients that progressed to an incurable setting and those who did not.

Lastly, only Dutch patients were included in the current study. The available treatment options in the Netherlands are comparable to other western countries, but how our results relate to other countries remains to be investigated.

A strength of our study is its nationwide base and a mostly unbiased patient selection process. Inclusion did not depend on the region, place of death, type of health insurance plan, or hospital in which a patient was treated, and therefore, our results are less prone to selection bias.

## 5. Conclusions

This nationwide observational study showed a relatively low prevalence of chemotherapy use and ICU admission in the last three months before death in Dutch patients deceased with advanced gastric or esophageal cancer. Oncologists working in high-volume hospitals may be better equipped in selecting patients most likely to benefit from chemotherapy in the last months of life. If our results can be replicated in other studies, they support an increased effort to exchange knowledge and experience between high- and low-volume hospitals.

## Figures and Tables

**Table 1:** Descriptive characteristics of included patients.

Demography and Cancer Characteristics	Findings
Men—no. (%)	2676 (71.4%)
Age at time of death—mean (SD)	71.4 (11.1)
Esophageal cancer—no. (%)	2448 (65.3%)
Gastric cancer—no. (%)	1300 (34.7%)
Comorbidities	
Hypertension—no. (%)	2413 (64.4%)
Diabetes—no. (%)	766 (20.4%)
COPD—no. (%)	515 (13.7%)
Clinical descriptives	
Radiation therapy in the final 3 months—no. (%)	739 (19.7%)
Deceased in hospital—no. (%)	865 (23.1%)

**Table 2:** Prevalence of intensive care unit (ICU) admission and chemotherapy in the months before death.

Outcome of Interest	3 Months before Death			Final Month before Death		
	Patients (no.)	Population (no.)	%	Patients (no.)	Population (no.)	%
ICU admission	209	3748	5.6%	156	3748	4.2%
						
Chemotherapy	795	3748	21.2%	299	3748	8.0%
Chemotherapy within subpopulations						
With previous chemotherapy	488	1017	48.0%	191	1216	15.7%
Without previous chemotherapy	307	2731	11.2%	108	2532	4.3%

ICU: intensive care unit; no.: number.

**Table 3:** Correlation of hospital number experience per indicator and ICU admission or chemotherapy use before death.

Outcome of Interest	3 Months before Death		Final Month before Death		
	Correlation r (Weighted)	Significance *p* (Two-Tailed)	Correlation r (Weighted)	Significance p (Two-Tailed)	Institutions (no.)
ICU admission	0.18	0.11	0.20	0.07 *	81
Chemotherapy	−0.12	0.29	−0.23	0.04 **	81
Chemotherapy within subpopulations					
With previous chemotherapy	−0.12	0.30	−0.18	0.12	75
Without previous chemotherapy	−0.21	0.06 *	−0.23	0.03 **	81

ICU: intensive care unit; no.: number; * *p* < 0.10; ** *p* < 0.05 (two-tailed); correlations are weighted for number of observations.

**Table 4:** Correlation of treatment with volume of patients with chemotherapy per healthcare institute.

Outcome of Interest	3 Months before Death		Final Month before Death		
	Correlation r (Weighted)	Significance *p* (Two-Tailed)	Correlation r (Weighted)	Significance *p* (Two-Tailed)	Institutions (no.)
ICU admission	0.02	0.84	0.07	0.55	81
Chemotherapy	−0.01	0.92	−0.07	0.55	81
Chemotherapy within subpopulations					
With previous chemotherapy	−0.23	0.04 **	−0.11	0.33	75
Without previous chemotherapy	−0.05	0.67	−0.08	0.46	81

ICU: intensive care unit; no.: number; ** *p* < 0.05 (two-tailed); correlations are weighted for number of observations.

## Data Availability

The data that support the findings of this study are subject to third party restrictions (Vektis), please contact J. Reitsma for details and requests for data sharing.

## Supplementary Materials

The following are available online at https://www.mdpi.com/2072-6694/13/1/145/s1, Table S1: Diagnoses codes used to identify patients with gastric or esophageal cancer, Table S2: Care activity codes corresponding with surgical resections, Table S3: Care activity codes corresponding with radiation, Table S4: Care activity codes corresponding with chemotherapy, Table S5: Care activity codes corresponding with endoscopic procedures, Table S6: Care activity codes corresponding ICU admissions.

## References

[1] Cancer Today—Estimated Number of New Cases in 2018. https://gco.iarc.fr/today/online-analysis-table.

[2] Cancer Today—Stomach Cancer Fact Sheet. https://gco.iarc.fr/today/data/factsheets/cancers/7-Stomach-fact-sheet.pdf.

[3] Cancer Today—Esophageal Cancer Fact Sheet. https://gco.iarc.fr/today/data/factsheets/cancers/6-Oesophagus-fact-sheet.pdf.

[4] Van Putten M., De Vos-Geelen J., Nieuwenhuijzen G.A.P., Siersema P.D., Lemmens V.E.P.P., Rosman C., Van Der Sangen M.J.C., Verhoeven R.H.A. (2018). Long-term survival improvement in oesophageal cancer in the Netherlands. Eur. J. Cancer.

[5] Nelen S.D., Verhoeven R.H.A., Lemmens V.E.P.P., De Wilt J.H.W., Bosscha K. (2017). Increasing survival gap between young and elderly gastric cancer patients. Gastric Cancer.

[6] Van Kleef J.J., Ter Veer E., Boorn H.G.V.D., Schokker S., Ngai L.L., Prins M.J., Mohammad N.H., Van De Poll-Franse L.V., Zwinderman A.H., Van Oijen M.G.H. (2019). Quality of Life During Palliative Systemic Therapy for Esophagogastric Cancer: Systematic Review and Meta-Analysis. J. Natl. Cancer Inst..

[7] Wright A.A., Zhang B., Ray A., Mack J.W., Trice E., Balboni T., Mitchell S.L., Jackson V.A., Block S.D., Maciejewski P.K. (2008). Associations between end-of-life discussions, patient mental health, medical care near death, and caregiver bereavement adjustment. JAMA.

[8] Gade G., Venohr I., Conner D., McGrady K., Beane J., Richardson R.H., Williams M.P., Liberson M., Blum M., Della Penna R. (2008). Impact of an Inpatient Palliative Care Team: A Randomized Controlled Trial. J. Palliat. Med..

[9] Brumley R., Enguidanos S., Jamison P., Seitz R., Morgenstern N., Saito S., McIlwane J., Hillary K., Gonzalez J. (2007). Increased Satisfaction with Care and Lower Costs: Results of a Randomized Trial of In-Home Palliative Care. J. Am. Geriatr. Soc..

[10] Maetens A., Beernaert K., De Schreye R., Faes K., Annemans L., Pardon K., Deliens L., Cohen J. (2019). Impact of palliative home care support on the quality and costs of care at the end of life: A population-level matched cohort study. BMJ Open.

[11] Merchant S.J., Lajkosz K., Brogly S.B., Booth C.M., Nanji S., Patel S.V., Baxter N.N. (2017). The Final 30 Days of Life: A Study of Patients with Gastrointestinal Cancer in Ontario, Canada. J. Palliat. Care.

[12] Tramontano A.C., Nipp R., Kong C.Y., Yerramilli D., Gainor J.F., Hur C. (2018). Hospice use and end-of-life care among older patients with esophageal cancer. Health Sci. Rep..

[13] Mohammad N.H., Bernards N., Van Putten M., Lemmens V.E.P.P., Van Oijen M.G.H., Van Laarhoven H.W.M. (2017). Volume-outcome relation in palliative systemic treatment of metastatic oesophagogastric cancer. Eur. J. Cancer.

[14] Kempf E., Tournigand C., Rochigneux P., Aubry R., Morin L. (2017). Discrepancies in the use of chemotherapy and artificial nutrition near the end of life for hospitalised patients with metastatic gastric or oesophageal cancer. A countrywide, register-based study. Eur. J. Cancer.

[15] Hong J.H., Rho S.-Y., Hong Y.S. (2013). Trends in the Aggressiveness of End-of-Life Care for Advanced Stomach Cancer Patients. Cancer Res. Treat..

[16] Dijksterhuis W.P.M., Verhoeven R.H.A., Meijer S.L., Slingerland M., Mohammad N.H., De Vos-Geelen J., Beerepoot L.V., Van Voorthuizen T., Creemers G.-J., Van Oijen M.G.H. (2020). Increased assessment of HER2 in metastatic gastroesophageal cancer patients: A nationwide population-based cohort study. Gastric Cancer.

[17] Evans N., Pasman H.R., Alonso T.V., Block L.V.D., Miccinesi G., Van Casteren V., Donker G., Bertolissi S., Zurriaga O., Deliens L. (2013). End-of-Life Decisions: A Cross-National Study of Treatment Preference Discussions and Surrogate Decision-Maker Appointments. PLoS ONE.

[18] Evans N., Costantini M., Pasman H.R., Block L.V.D., Donker G.A., Miccinesi G., Bertolissi S., Gil M., Boffin N., Zurriaga O. (2014). End-of-Life Communication: A Retrospective Survey of Representative General Practitioner Networks in Four Countries. J. Pain Symptom Manag..

[19] Bestvina C.M., Wroblewski K.E., Daly B., Beach B., Chow S., Hantel A., Malec M., Huber M.T., Polite B.N. (2018). A Rules-Based Algorithm to Prioritize Poor Prognosis Cancer Patients in Need of Advance Care Planning. J. Palliat. Med..

[20] Turpin M.H., Meyers E.A., Fugelsang J.A., Friedman O., Białek M. (2019). Sunk Cost Bias and Withdrawal Aversion. Am. J. Bioeth..

[21] Meltzer D., Manning W.G., Morrison J., Shah M.N., Jin L., Guth T., Levinson W. (2002). Effects of Physician Experience on Costs and Outcomes on an Academic General Medicine Service: Results of a Trial of Hospitalists. Ann. Intern. Med..

[22] Birkmeyer J.D., Dimick J.B. (2009). Understanding and Reducing Variation in Surgical Mortality. Annu. Rev. Med..

[23] Chan B.A., Larkins S.L., Evans R., Watt K., Sabesan S. (2015). Do teleoncology models of care enable safe delivery of chemotherapy in rural towns?. Med. J. Aust..

[24] Salami A.C., Barden G.M., Castillo D.L., Hanna M., Petersen N.J., Davila J.A., Naik A.D., Anaya D.A. (2015). Establishment of a Regional Virtual Tumor Board Program to Improve the Process of Care for Patients with Hepatocellular Carcinoma. J. Oncol. Pr..

[25] Indini A., Aschele C., Cavanna L., Clerico M., Daniele B., Fiorentini G., Fioretto L., Giordano M., Montesarchio V., Ortega C. (2020). Reorganisation of medical oncology departments during the novel coronavirus disease-19 pandemic: A nationwide Italian survey. Eur. J. Cancer.

[26] Van Erning F.N., Van Steenbergen L.N., Broek W.V.D., Rutten H.J.T., Lemmens V.E.P.P. (2013). No difference between lowest and highest volume hospitals in outcome after colorectal cancer surgery in the southern Netherlands. Eur. J. Surg. Oncol. EJSO.

[27] Jonker F.H.W., Hagemans J.A.W., Verhoef C., Burger J.W.A. (2017). The impact of hospital volume on perioperative outcomes of rectal cancer. Eur. J. Surg. Oncol. EJSO.

[28] Vos M., Blaauwgeers H.G.T., Ho V.K.Y., Van Houdt W.J., Van Der Hage J.A., Been L.B., Bonenkamp J.J., Bemelmans M.H.A., Van Dalen T., Haas R.L. (2019). Increased survival of non low-grade and deep-seated soft tissue sarcoma after surgical management in high-volume hospitals: A nationwide study from the Netherlands. Eur. J. Cancer.

