# Supplementary material for: Use of Palliative Chemotherapy and ICU Admissions in Gastric and Esophageal Cancer Patients in the Last Phase of Life: A Nationwide Observational Study
Source: Cancers (Basel). 2021 Jan 5;13(1):145. doi: 10.3390/cancers13010145 (PMC7794997; doi:10.3390/cancers13010145)
Supplement: Supplementary file 1 [file cancers-13-00145-s001.pdf]

# Supplementary Materials:

The detailed definitions used on the basis of the Dutch medical claims data are given below. Table 1 provides codes of diagnoses. A patient needs at least one diagnosis code to successfully claim medical costs, but he or she can have multiple codes. All care activities that a patient undergoes are linked to a diagnosis code. Different specialties use different treatments with corresponding treatment codes. Therefore each specialty uses a unique diagnosis code and this can result in multiple diagnosis codes per patient for one diagnosis (one for each specialty). Patients were initially selected based on diagnosis codes.

Table 2 to 5 show the codes for care activities as described in the Dutch medical claims data, with which further selection of patients was done. These care activities are grouped by surgery (table 2), radiation (table 3), chemotherapy (table 4) and endoscopic treatments (table 5). In table 6 the care activities corresponding with ICU admissions are listed.

In all tables both the Dutch and translated English description are provided to enhance transparency and reproducibility.

**Table S1.** Diagnoses codes used to identify patients with gastric or esophageal cancer.

| Specialty name | Diagnosis code | Diagnosis description (Dutch) | Diagnosis description (English) |
|---|---|---|---|
| Surgery | 319 | Maligne neoplasma oesofagus / cardia | Cancer of esophagus and cardia of stomach |
| Surgery | 346 | Maligne neoplasma maag, exclusief cardia | Stomach cancer (excluding tumors of the cardia) |
| Internal medicine | 904 | Maligniteit oesofagus /cardia | Cancer of esophagus and cardia of stomach |
| Internal Medicine | 914 | Maligniteit maag (exclusief cardia) | Stomach cancer (excluding tumors of the cardia) |
| Gastroenterology | 307 | Oesofagus/cardia maligniteit (esophageal cancer) | Cancer of esophagus and cardia of stomach |
| Gastroenterology | 407 | Maagcarcinoom, exclusief cardiacarcinoom | Stomach cancer (excluding tumors of the cardia) |

**Table S2.** Care activity codes corresponding with surgical resections.

| Care activity | Description (Dutch) | Description (English) |
|---|---|---|
| 034432 | Maagresectie | Gastrectomy |
| 034440 | Totale maagresectie, thoracaal of abdominaal | Gastrectomy, thoracic or abdominal approach |
| 034436 | Reconstructieve operatie aan de resectiemaag volgens bijvoorbeeld Henley of Roux-Y | Reconstructive surgical operation on resected stomach (e.g. according Henley or Roux-en-Y) |
| 034322 | Resectie van oesophaguscarcinoom | Resection of esophageal cancer |
| 034323 | Oesophagusresectie, open procedure (zie 034324 voor thoracoscopisch). | Resection of esophagus, open approach (see 034324 for thoracoscopic approach) |
| 034324 | Thoracoscopische oesophagusresectie (zie 034323 voor open procedure). | Resection of esophagus, thoracoscopic approach (see 034323 for open approach) |
| 034342 | Oesophagusresectie met colonimplantatie (met jejunuminterpositie zie 034398, met gastric pull-up zie 034399). | Resection of esophagus with colon implantation (see 034398 for procedure with jejunal interposition and 034399 for procedure with gastric pull up) |

**Table S3.** Care activity codes corresponding with radiation.

| Care activity | Description (Dutch) | Description (English) |
|---|---|---|

| | | |
|---|---|---|
| 090791 | Een bestralingsfractie | One fraction of radiation |
| 090796 | Stereotactische bestraling (exclusief stereotactische protonenbestraling, zie 090832 en 090833). | Stereotactic radiation (excluding stereotactic proton radiation; see 090832 and 090833) |
| 090797 | Intensieve bestraling | Intensive radiation |

**Table S4.** Care activity codes corresponding with chemotherapy.

| Care activity | Description (Dutch) | Description (English) |
|---|---|---|
| 39141 | Verstrekking chemotherapie per infuus of per injectie bij niet-gemetastaseerde tumoren. | Delivery of chemotherapy via intravenous dwelling catheter or via injection, in non-metastasized cancer |
| 39142 | Verstrekking chemotherapie per infuus of per injectie bij gemetastaseerde tumoren. | Delivery of chemotherapy via intravenous dwelling catheter or via injection, in metastasized cancer |
| 39143 | Verstrekking chemotherapie per infuus of per injectie bij acute leukemie. | Delivery of chemotherapy via intravenous dwelling catheter or via injection, in acute leukemia |
| 39144 | Verstrekking chemotherapie per infuus of per injectie bij niet-oncologische diagnosen. | Delivery of chemotherapy via intravenous dwelling catheter or via injection, in non-oncologic diagnosis |
| 39145 | Verstrekking chemo-immunotherapie per infuus of per injectie. | Delivery of chemotherapy via intravenous dwelling catheter or via injection |
| 39958 | Hyperthermische intraperitoneale chemotherapie (HIPEC) in combinatie met cytoreductie. | Hyperthermic intraperitoneal chemotherapy (HIPEC) combined with cytoreduction |
| 34730 | Adjuvante hyperthermische intraperitoneale chemotherapie (HIPEC; voor HIPEC in combinatie met cytoreductie zie 039958). | Adjuvant hyperthermic intraperitoneal chemotherapy (HIPEC; for HIPEC combined with cytoreduction see 039958) |

**Table S5.** Care activity codes corresponding with endoscopic procedures.

| Care activity | Description (Dutch) | Description (English) |
|---|---|---|
| 34640 | Endoscopische mucosectomie | Endoscopic mucosal resection |

**Table S6.** Care activity codes corresponding ICU admissions.

| Care activity | Description (Dutch) | Description (English) |
|---|---|---|
| 190153 | IC-dag licht (valid until Dec, 31st, 2017) | One day of ICU admission, light (valid until December the 31st of 2017) |
| 190154 | IC-dag middel (valid until Dec, 31st, 2017) | One day of ICU admission, moderate (valid until December the 31st of 2017) |
| 190155 | IC-dag zwaar (valid until Dec, 31st, 2017) | One day of ICU admission, heavy (valid until December the 31st of 2017) |
| 190157 | IC-dag type 1* (valid from Jan, 1st, 2018) | One day of ICU admission type 1 (valid from January the 1st of 2018) |
| 190158 | IC-dag type 2* (valid from Jan, 1st, 2018) | One day of ICU admission type 2 (valid from January the 1st of 2018) |

** Type 1 and Type 2 correspond to different ways that the ICU is processed in registrations and declarations. There is no clear relation with light/moderate/heavy ICU.*
